# Construction of carbon nanostructures using the sodium cocoyl glycinate@NaCl system

**DOI:** 10.1039/d4ra05655h

**Published:** 2024-09-04

**Authors:** Xiaofei Kang, Haichao Li, Xiaobo Yang, Zezhong Lin

**Affiliations:** a Qinghai Nationalities University China 18291780747@163.com lihaichao@vip.163.com yang_1101979@163.com 102021013@tju.edu.cn

## Abstract

Carbon nanomaterials are widely used in many fields due to their unique properties. However, the novel production of carbon nanostructures is still a research challenge. Here, the self-assembly system of the anion surfactant sodium cocoylonate (SC)@NaCl has successfully produced a cube-shaped carbon nanoframework. The surface morphology, graphitization degree, elemental composition, surface chemical state, formation mechanism and photoluminescence properties of the carbon nanomaterials were further investigated. The results show that the surfactant–salt system is a novel and environmentally friendly method for producing nanostructures.

## Abstract 2

Carbon nanomaterials are widely used in many fields due to their unique properties.

## Graphical abstract

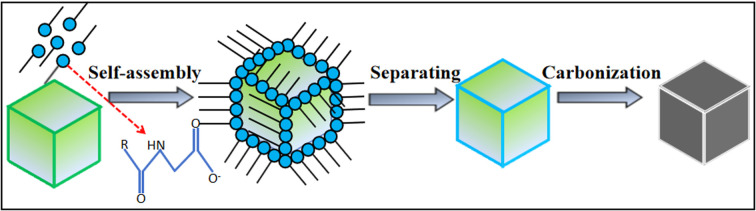

## 1. Introduction

Carbon is an element that captivates people because it can form hybridizations of sp, sp^2^, and sp^3^ electron orbitals, leading to a variety of compounds and structures with diverse characteristics. Carbon is the foundation of organic life, but it also forms many inorganic allotropes and forms. Graphite, diamond, fullerenes, and nanotubes have attracted significant attention due to their diverse properties, which are extensively applied in nearly every field of human activity, exhibiting excellent mechanical, electrical, thermal, and optical performance. Naturally, carbon chemistry, especially the chemistry of graphitic carbon, is one of the most studied fields in chemistry.^1–3^ Over the past 20 years, there have been significant advancements in carbon nanostructures. However, there is still much room for further research. Researchers must transition laboratory findings to an industrial scale.^4^ With continuous technological development, scientists have delved into the study of nanoscale carbon materials, reporting on these carbon nanomaterials: for example, fullerenes,^5^ carbon nanotubes,^6^ graphene,^7^ carbon quantum dots,^8^ carbon fibers, and three-dimensional carbon materials. There are various methods for preparing carbon nanomaterials, common ones include Chemical Vapor Deposition (CVD), arc discharge methods, and laser ablation. In addition to the above methods, there are techniques for preparing carbon nanotube films through dry and wet processes, mainly used for assembling flexible electronic devices and studying their performance applications. However, the discovery and development of new carbon nanostructures is a complex process that integrates scientific prediction, technological innovation, and application exploration. In promoting their sustainable production and commercialization, it is essential not only to consider production efficiency and cost control but also to pay attention to environmental impacts and health risks while overcoming various technical and market challenges.

For example, based on previous reports in the literature, carbon nanostructures with different morphologies were prepared using the cationic surfactants dodecyldimethylbenzylammonium chloride (DDBAC)@NaCl^9^ and dodecyldimethyltrimethylammonium bromide DTAB@NaCl,^10^ respectively. The common feature of these manufacturing methods is the use of a surfactant and inorganic salt systems. Therefore, in this work, we report a novel environmentally friendly and cost-effective method for the preparation of carbon nanomaterials, *i.e.*, a surfactant@salt self-assembly strategy, for the synthesis of size-tunable carbon nanoframes. The surfactant is a type of lipophilic and hydrophilic amphiphilic molecule. This type of molecule is located in the aqueous solution system (including the surface, interface) relative to the aqueous medium and adopts a unique directional arrangement and forms a certain ordered structural organization.^11^

**Fig. 1:**
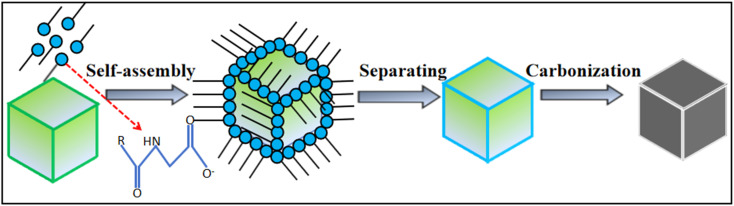
Schematic diagram of the formation mechanism of SC@NaCl carbon nanoframes.

Sodium cocoyl glycinate is one of the amino acid surfactants, and is an amino acid-based green surfactant synthesized by a chemical reaction using glycine and fatty acids of natural origin as raw materials. The raw material is part of the biological body, renewable, easy to biodegrade and of low toxicity. Therefore, it has great application prospects in food, medicine, cosmetics and other industries.^12,13^ In addition, SC has a low surface tension and optimal surface activity at a pH of medium alkaline.^14^ Therefore, we used selected ultrapure water as a solvent to dissolve and formulate SC into a solution and used SC as a carbon source. At the same time, the inexpensive inorganic salt NaCl was introduced as a template, and NaCl crystals were used to separate the ordered aggregates of SC during the carbonization process. NaCl acts as a separator and air barrier, which eliminates the need for the carbonization process to be protected by the passage of noble gases, and finally, carbon nanoframes with cubic structure have been successfully obtained, and the NaCl can be recycled after recovery through evaporation and crystallization. To enable the complete aggregation of SC on the surface of NaCl crystals through self-assembly, the carbon nanoframes were fabricated by carbonization and washing at high temperature to ensure that SC fully replicates the cubic structure of NaCl crystals, which is expected. The design purpose of this work is in Fig. 1. It is worth noting that this method is more environmentally friendly and economical compared to the traditional method because it does not require the addition of catalysts, has mild conditions, is simple and does not release toxic gases. However, this method currently represents a challenge in the large-scale production of high-quality and highly pure carbon nanostructures.

## 2. Experimental

### 2.1 Materials and their details

SC (30% aqueous solution) was purchased from Shanghai Yuanye Biotechnology Co. It is widely used in cosmetics and toiletries with low skin irritation, low toxicity and unknown long-term effects. SC is usually in solid form and can be a white or yellowish powder or granules. Sodium chloride (analytically pure grade) was purchased from Tianjin Damao Chemical Reagent Factory. Excessive intake of sodium chloride by the human body can lead to certain risks of diseases. Pure sodium chloride usually consists of colorless and transparent cubic crystals, but due to the presence of impurities it often appears white or has a certain color. The water used throughout the experiment was ultrapure water.

### 2.2 Preparation of carbon nanoframes

Using SC as the carbon source, NaCl crystals are employed to separate the ordered aggregates of SC during the carbonization process. Specifically, the precursor (SC@NaCl) is prepared by combining segregation and carbonization to guide the self-assembly of the “core” (SC) and “shell” (NaCl) into the SC@NaCl precursor. The “shell” part can be dissolved in water after carbonization and detached from the “guided core”, thereby obtaining carbon nanomaterials with controllable particle size. The specific operation is as follows: in the solvent water, the critical micelle concentration (CMC) value of SC is 3.86 × 10^−3^ mol L^−1^.^14^ 100 mL of SC solutions (0.5CMC, 1CMC, 5CMC, and 10CMC) were prepared separately to which 80 g of NaCl was added and stirred with a magnetic stirrer at 180 °C (300 rpm) until all the water evaporated, so that SC was completely wrapped in the surface of NaCl to complete the self-assembly process, *i.e.*, the SC@NaCl system. The precursor was placed in a box-type resistance furnace and heated to 600 °C for carbonization at a rate of 5 °C min^−1^ for 1 h until it was restored to room temperature to complete the carbonization process, and carbon nanomaterials were prepared. NaCl is washed away with ultrapure water, and the remaining “nucleus” is the prepared carbon nanoframe. It is worth noting that the evaporation and crystallization of the filtrate to recover NaCl can be recycled.

### 2.3 Characterization

Raman spectra were measured by an inVia Reflex laser microscope confocal Raman spectrometer (Renishaw, UK). An argon-ion laser was used as the excitation light source, and the excitation wavelength was set to *λ* = 532 nm. The spectral range of the acquisition was from 100 to 3000 cm^−1^. The crystalline structure parameters of the samples were determined using a D/MAX-B type X-ray diffractometer from RIKEN, Japan. Cu target radiation (*λ* = 0.154056 nm) and scanned the sample at a scanning speed of 4° min^−1^ in the range of 2*θ* = 15°–60°. The morphology of carbon nanomaterials was observed by SEM (ZEISS GeminiSEM 500, Germany). TEM images were obtained with a JEM-2100F transmission electron microscope (JEOL, Japan) at 200 kV. The particle size distribution of the samples was measured by the DLS method (OTSUKA FRAR-1000, Japan). The fluorescence spectra were recorded by an F-4600 fluorescence spectrometer (Hitachi, Japan). The emission spectra were recorded in the range of 300–800 nm at the excitation wavelengths of 300, 320, 340, 360, 380, 400 and 420 nm. FTIR detection was performed using Thermo Fisher NICOLET IS5. Elemental composition analysis in atomic percent was conducted using energy-dispersive spectroscopy (EDS) integrated with the scanning electron microscope, with elemental mapping carried out for all the components of the prepared materials.

## 3. Results and discussion

**Fig. 2:**
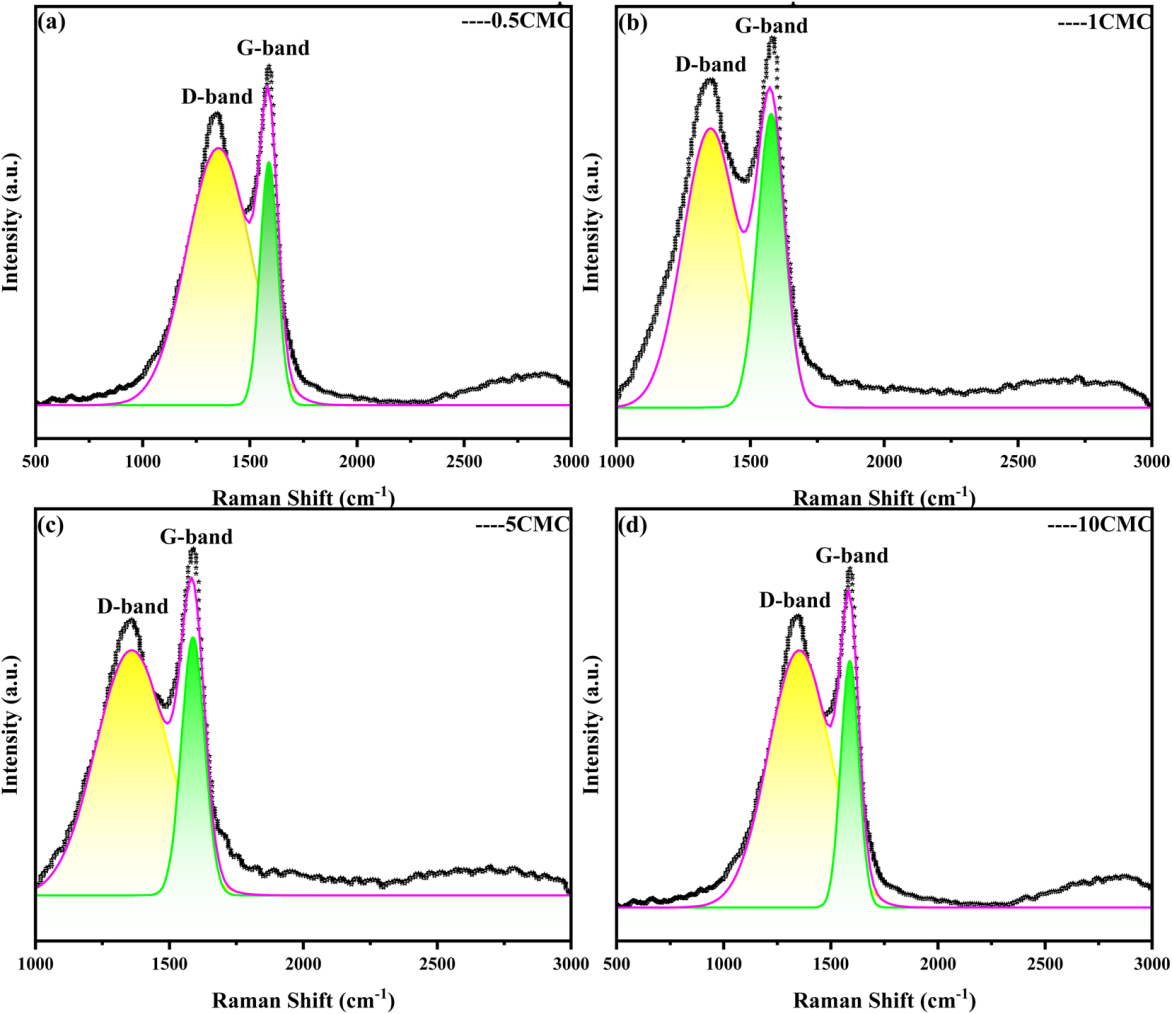
Raman spectra of SC@NaCl carbon nanoframes.

Raman spectrum is an effective method for analyzing the structure of carbon material.^15^ This is a scattering spectrum analysis method that can be used to characterize structural features such as defects and disorder of the nanoporous carbon structure. The Raman spectrum obtained at four different concentrations is shown in Fig. 2. Raman spectra of carbon nanostructures show the G and D bands of typical graphite structures. The G band (about 1590 cm^−1^) is the sp^2^ hybridized in-plane stretching vibration of carbon atoms, which is generated by the stretching motion of sp^2^ atom pairs in carbon rings or carbon chains and symbolizes the degree of graphitization of the materials. And the D band (at around 1360 cm^−1^) is the disordered activation band.^16,17^ The ratio of the intensity of the D and G bands in Raman spectroscopy (*I*_D_/*I*_G_ ratio) is often used as a measure of the degree of graphitization of a sample and as a measure of the quality of the carbon nanostructures produced. A smaller *I*_D_/*I*_G_ ratio corresponds to fewer defects.^18^ The relatively strong and narrow G-belt means relatively high graphite content, which is conducive to SC@NaCl materials. However, the D-belt is very wide, suggesting that the sample has an amorphous structure.^19^ The *I*_D_/*I*_G_ obtained by the 4 different concentrations is 0.86, 0.89, 0.80, 0.84 in Table 1, and the *I*_D_/*I*_G_ value of 4 samples is very close to each other. At 5CMC, the *I*_D_/*I*_G_ value is the lowest, indicating that the carbon nanostructures obtained by carbonization at 5CMC are the smallest and the crystal structure is more complete, indicating that the graphite of the carbon content in of the sample is highest. It is worth noting that the calculation results show that the *I*_D_/*I*_G_ values of 0.5CMC, 1CMC, 5CMC and 10CMC are less than 1. This proves the high graphite content of their carbon framework.^20^

**Table 1:** Raman parameters (peak position, peak intensity and *I*_D_/*I*_G_ ratio) obtained from the peak fit of the Raman spectra of each sample

	0.5CMC	1CMC	5CMC	10CMC
D peak (cm^−1^)	1347.8	1349.6	1360.52	1353.95
D peak intensity (cm^−1^)	87.88	530.98	607.228	913.803
G peak (cm^−1^)	1590.11	1582.42	1589.64	1583.99
G peak intensity (cm^−1^)	102.457	599.272	761.865	1087.286
*I*_D_/*I*_G_	0.86	0.89	0.80	0.84

**Fig. 3:**
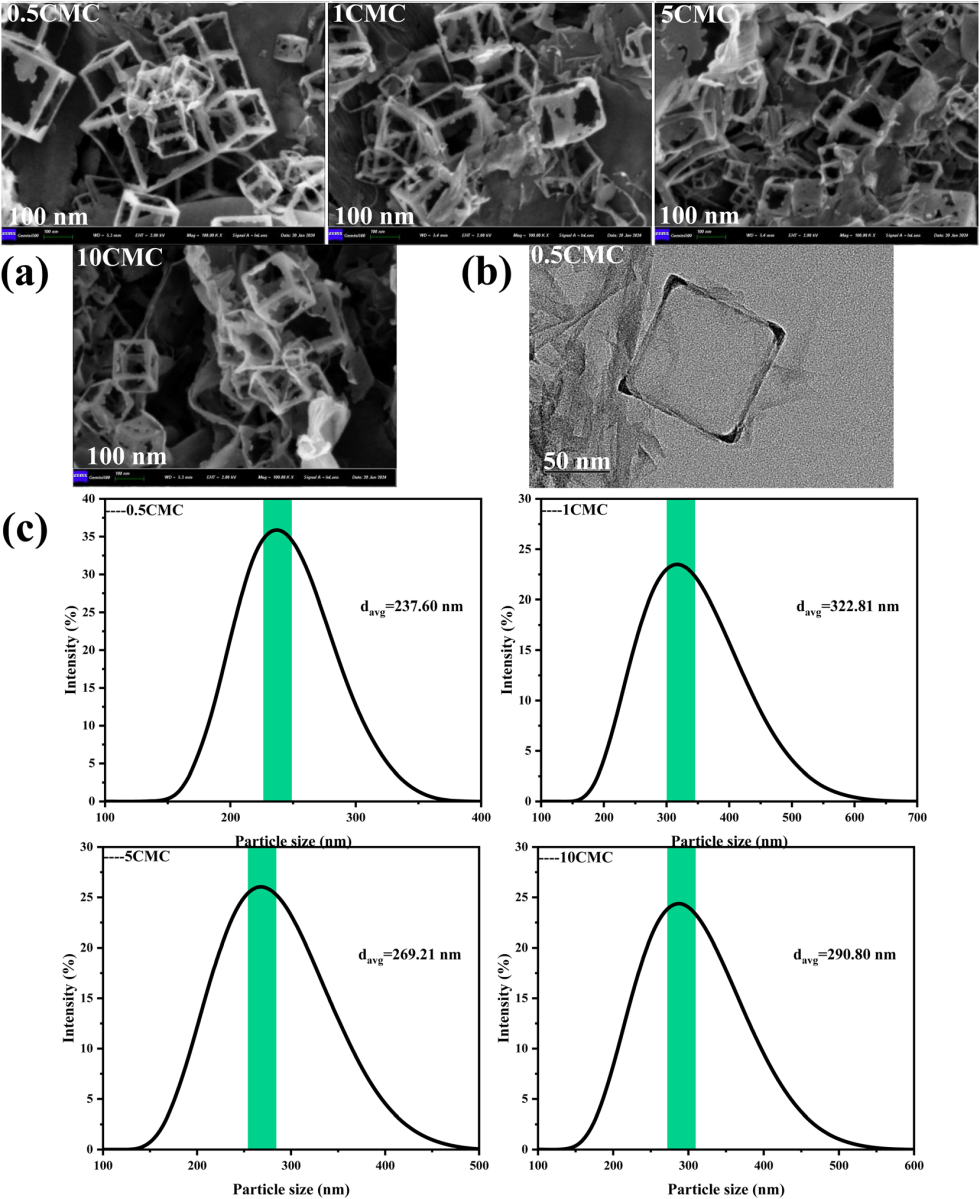
Synthesis of carbon nanoframes by the SC@NaCl self-assembly strategy. (a) Carbon nanoframes shown in representative SEM images at SC concentrations of 0.5, 1, 5 and 10CMC. (b) Carbon nanoframes shown in representative TEM images at SC concentrations of 0.5CMC. (c) DLS curves of carbon nanoframes at different SC concentrations obtained after pyrolysis.

Scanning electron microscope (SEM) is an instrument that provides high-resolution images of the microstructure of a material. Scanning electron microscopy shows in Fig. 3(a) that the size and shape of the carbon nanoframework are very uniform and regular. This is mainly due to the fact that SCG reproduces the cubic morphology of NaCl crystals during the self-assembly process, while the high temperature carbonization process maintains this dispersion and cubic structure, which is also consistent with the design of this experiment. Fig. 3(b) shows the TEM image of the carbon nanostructures obtained at a concentration of 0.5CMC. Transmission electron microscopy (TEM) was used to observe the morphology of the product and the development of the internal structure of the sample. Transmission electron microscopy shows the evolution of the internal structure of the sample as well as further details of the individual carbon nanoframes. At 0.5CMC, the surfactant did not reach CMC and was present as a monomer. As can be seen from the TEM images, the 0.5CMC samples formed rectangular, regularly layered carbon nanostructures.^21^ 0.5CMC indicates a particle of about 50 nm at a temperature of 600 °C. SC was involved in the crystal growth process, but contrary to previous reports in the literature, the surfactant is not concentrated on the surface of the crystal but is adsorbed at the edge of the crystal.^22^ Furthermore, the Dynamic Light Scattering (DLS) results show that the prepared carbon nanoframes have a uniform size, as shown in Fig. 3(c). At different concentrations (0.5CMC, 1CMC, 5CMC and 10CMC), the mean particle sizes of the carbon nanoframes were 237.60 nm, 269.21 nm, 322.81 nm and 290.80 nm, respectively. When the concentration of SCG increased from 0.5CMC to 1CMC, the particle size increased from 237.60 nm to 322.81 nm. This is because more surfactant molecules are involved in the formation of micelles when the CMC is close to or reaches the CMC,which leads to an increase in the particle size of the carbon nanoframes. However, when the concentration of SC was further increased to 5CMC and 10CMC, the average particle size was 269.21 nm and 290.80 nm, respectively, which decreased compared to 1CMC. This phenomenon may be due to the increase in surfactant concentration, so that the number of micelles increased but the size of individual micelles decreased. Therefore, the particle size of carbon nanoframes first increases and then decreases, which is closely related to the concentration of surfactant. Therefore, the size of carbon nanoframes can be effectively adjusted by adjusting the concentration of surfactants, which is of great importance in the field of materials science and drug delivery.

**Fig. 4:**
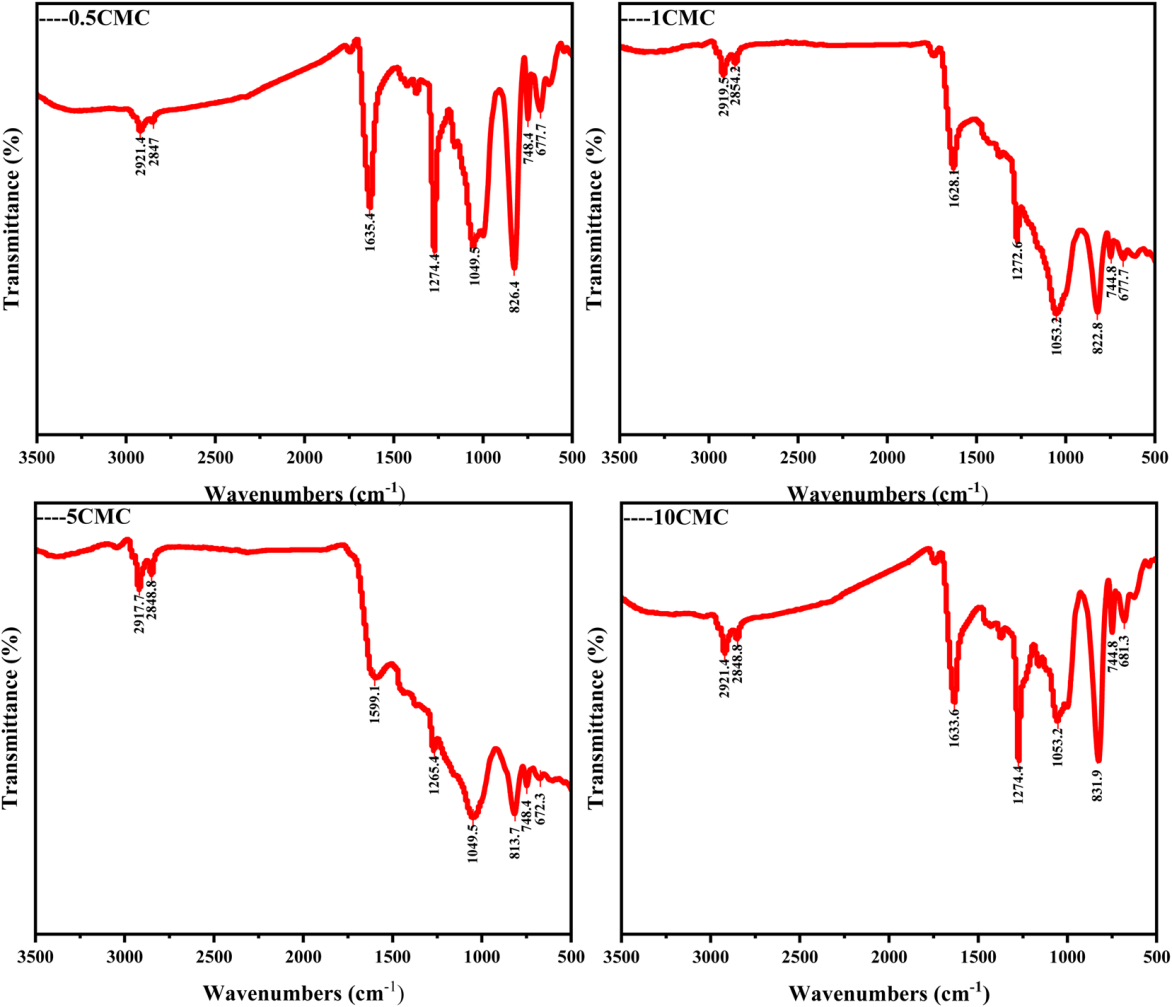
FTIR spectra of SC@NaCl carbon nanoframes.

In Fourier transform infrared spectroscopy (FTIR) analysis, different chemical bonds and functional groups absorb specific frequencies of infrared light and form characteristic peaks in the spectrum. The frequencies of these characteristic peaks (expressed in wavenumber cm^−1^) can help us identify and analyze the molecular structures in the samples. For this purpose, FTIR spectra were recorded and characteristic peaks of several functional groups were identified in Fig. 4. Carbon nanostructures contain C–O, C=O, C–H and other oxygen-containing functional groups all four samples.^23^ In particular, the C–H stretching peaks in the CH_2_ and CH_3_ groups appeared near 2920 cm^−1^,^24^ a wide peak near 1051 cm^−1^ can be attributed to the tensile vibration of C–O.^25^ The strong absorption peak near 1630 cm^−1^ corresponds to the C=O tensile vibration peak on the surface of carbon nanostructures.^26^ The peak value near 1270 cm^−1^ is related to the C–N tensile vibration mode.^26^ 820 cm^−1^ near the tip is consistent with the C–H bending vibration mode.^24^ FTIR data both show that the sample contains some O and N, which is a good explanation as to why the SC@NaCl carbon nanostructure is defective and an amorphous carbon structure.^27^

**Fig. 5:**
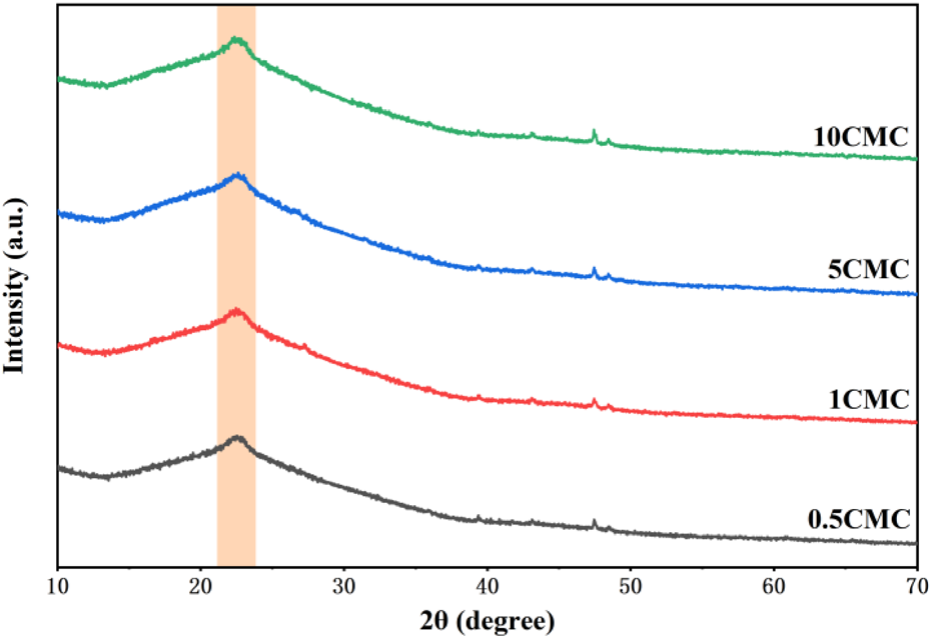
XRD pattern of SC@NaCl carbon nanostructures.

Further evidence of the extent of graphitization and defects in the SC@NaCl carbon nanostructures was obtained by powder X-ray diffraction (XRD). Fig. 5 shows the coexistence of diffraction peaks and diffraction curves for four groups of samples, indicating the presence of both crystalline and amorphous phase structures in the material.^28^ The differences in the characteristic peaks indicate differences in the crystal structure of the four sample groups.^29^ All four sample groups exhibit broader (002) diffraction peaks with asymmetric distribution. This indicates that the SC@NaCl samples are partially graphitized and contain a certain percentage of defects or amorphous carbon, which is consistent with the Raman spectroscopy results.

**Fig. 6:**
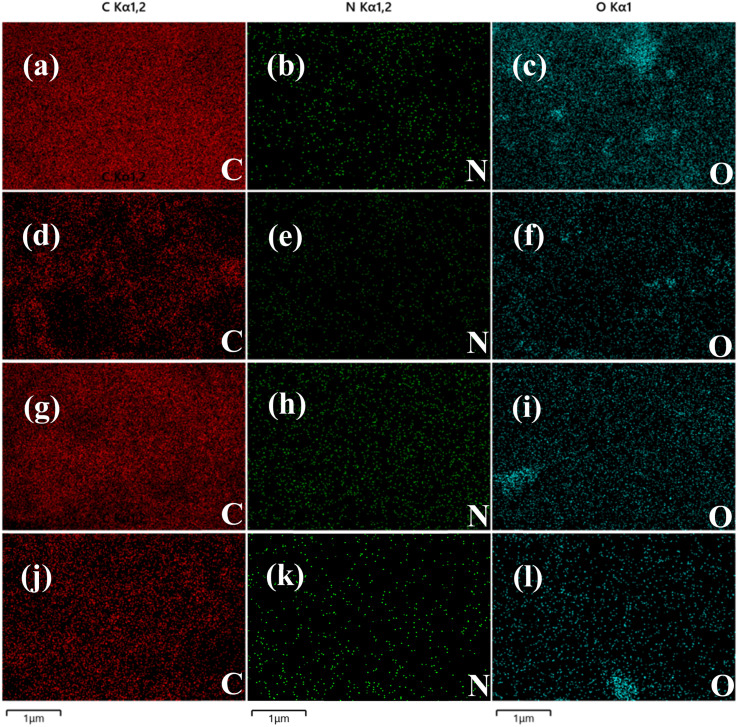
(a)–(c) EDS images of sample 0.5CMC. (d)–(f) EDS images of sample 1CMC. (g)–(i) EDS images of sample 5CMC. (j)–(l) EDS images of sample 10CMC.

In addition, the elemental composition was analyzed using energy spectrometry (EDS). The EDS results of carbon nanoframes fabricated using the SC@NaCl self-assembly system are shown in Fig. 6 and Table 2, respectively. The analysis confirmed that essential elements were present in all four samples and the element mapping diagrams correlated well between all elements in the samples. Table 2 summarizes the elemental composition of the carbon nanoframe structures. All samples contain detectable C, N and O. This is also consistent with the expected conjecture as the SC contains four elements, C, H, O and N, suggesting that these elements are present in a common compound. The EDS data suggests an even distribution of components for the four samples. In addition, we observed and did not observe impurity elements such as Na and Cl, suggesting that NaCl only remains on the surface of the carbon nanostructures and can be removed by washing with high-purity water. The good agreement with the XRD and FTIR results indicates that the carbon nanoframes have some defects.

**Table 2:** EDS analysis of elemental composition

Sample	Element	Wt (%)	At (%)
SC@NaCl (0.5CMC)	C	76.08	80.86
	N	0.55	0.50
	O	23.37	18.64
SC@NaCl (1CMC)	C	69.57	74.63
	N	7.56	6.95
	O	22.87	18.42
SC@NaCl (5CMC)	C	85.95	88.62
	N	4.57	4.04
	O	9.48	7.34
SC@NaCl (10CMC)	C	81.89	85.54
	N	2.27	2.03
	O	15.84	12.42

**Fig. 7:**
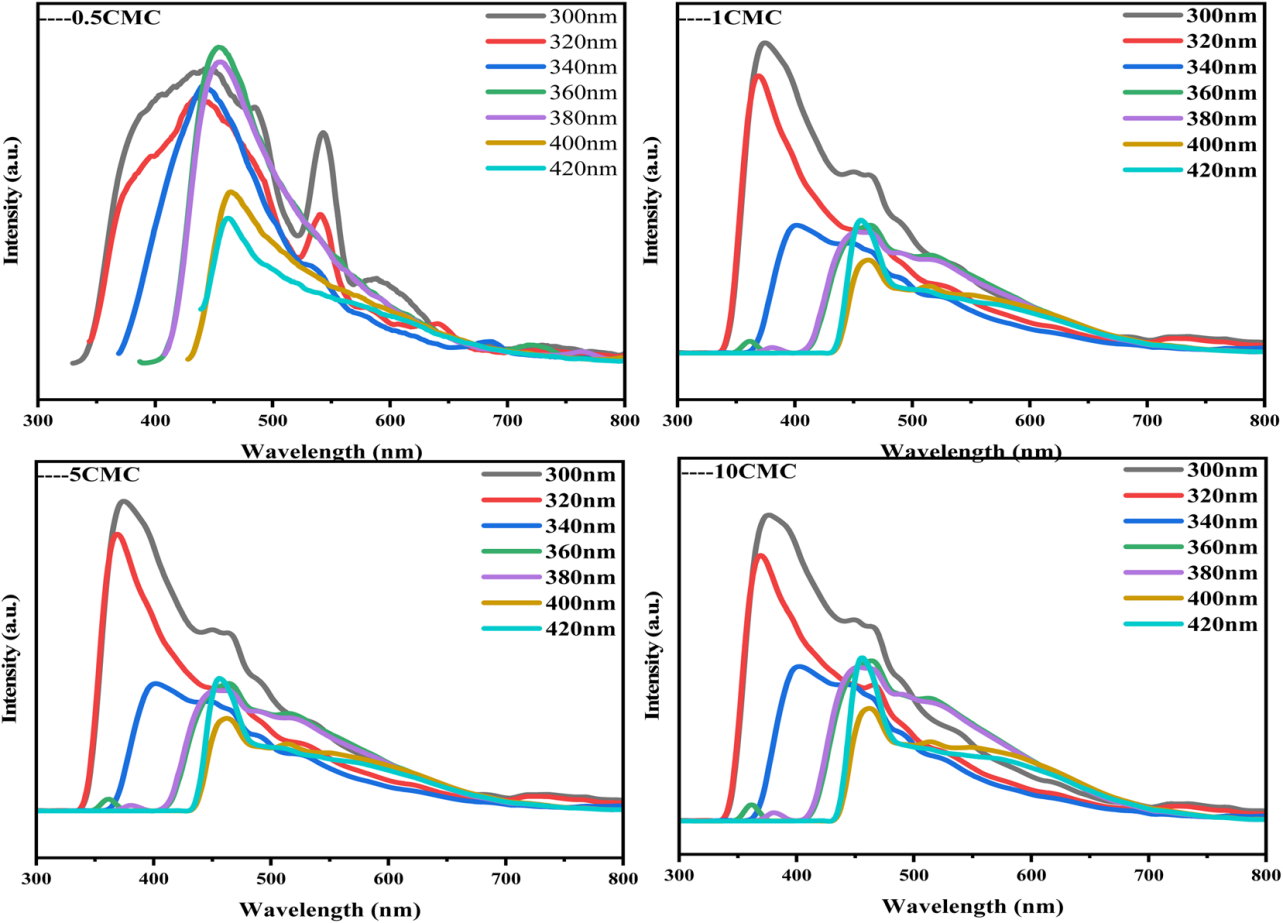
Excitation-dependent PL spectra of SC@NaCl when the excitation wavelength was varied from 300 nm to 420 nm.

Fluorescence spectroscopy (PL) is a technique for analyzing and detecting the luminescence properties of a substance. It is based on the principle that a substance emits fluorescence after absorbing light energy. Fig. 7 shows the PL spectra of four carbon nanoframes. All samples show significant fluorescence. Under 360 nm excitation, the maximum emission peak of 0.5CMC is concentrated near 450 nm, indicating its blue luminescence property. The possible reason for this difference is the different defective condition of the sample surface. In contrast, the strongest emission peaks of 1CMC, 5CMC and 10CMC can be observed at 380 nm, indicating that these three samples mainly exhibit violet emission behavior.^30^ Interestingly, 0.5CMC shows clear bimodal PL emission behavior at excitation from 300 nm to 320 nm.^31^ Furthermore, the emission wavelength of all four samples varies with the excitation wavelength and shows typical excitation-dependent fluorescence properties. In most cases, this property of carbon nanostructures can be due to the energy level distribution in their structures.^30,32,33^ This is because different energy levels exist in carbon nanoparticles, which may be due to the inhomogeneity of the particle size, different surface states, or doping by heteroatoms. When irradiated with excitation light of different energies, electrons can jump from different energy levels to the excited state and then release light of different wavelengths when returning to the ground state. Furthermore, we predict that the positions of functional groups on the surface of SC@NaCl carbon nanostructures can serve as energy trapping sites, which can trap excitons and perform jumps to generate fluorescence due to the presence of N and O atoms.^34^ Notably, the luminescence mechanism arises from a variety of reasons, including carbon excitons, emission traps, size effects, aromatic structures, surface groups, free zigzags, and edge defects.^35,36^ However, the exact mechanism of photoluminescence in carbon materials is not known.^37–47^ This SC@NaCl self-assembly successfully fabricated carbon nanomaterials with violet and dark blue emission. The properties of this new carbon nanoframe will have promising applications in molecular electronics, thermoelectric power generation, luminescence, building materials and medicine, *etc.*^48^

## 4. Conclusions

In conclusion, carbon nanomaterials with cubic structure were successfully prepared using the self-assembly strategy of SC@NaCl. Scanning electron microscopy (SEM) studies showed that the prepared carbon nanoframes had a uniform morphology. TEM and DLS results showed that the prepared carbon nanoframes could be adjusted to the size of the carbon nanostructures by changing the SC concentration. Raman spectra showed that the 5CMC carbon material had the highest degree of graphitization. FTIR, XRD and EDS data showed that the prepared carbon nanomaterials contained a certain amount of N and O, which further explains the disadvantages of the material. The formation mechanism of the carbon nanoframes also suggests that the shape and size of the micelles can be controlled by simply adjusting the concentration of the surfactant. PL analyzes show that the manufactured materials have specific optical properties (violet and deep blue light). In summary, the manufacturing method has the advantages of simplicity, environmental friendliness and low cost compared to previously described methods for synthesizing carbon nanomaterials. In addition, the method is expected to be widely applied to the carbonization of other surfactant@salt systems and provide new opportunities for the preparation of carbon nanomaterials with different functions.

## Conflicts of interest

We declare that we have no financial and personal relationships with other people or organizations that can inappropriately influence our work, there is no professional or other personal interest of any nature or kind in any product, service and/or company that could be construed as influencing the position presented in, or the review of, the manuscript entitled.
